# Facing a harsh climate: terrestrial plant functional strategies in a changing world

**DOI:** 10.1093/nsr/nwag416

**Published:** 2026-07-14

**Authors:** Jing Wang, Simon Pierce, Yuanzhi Li, Donghao Wu, Heng Huang, Josep Peñuelas, Bill Shipley, Chengjin Chu

**Affiliations:** State Key Laboratory of Biocontrol, School of Ecology, Shenzhen Campus of Sun Yat-sen University, Shenzhen 518107, China; Department of Agricultural and Environmental Sciences (DiSAA), University of Milan, Milan 20122, Italy; State Key Laboratory of Biocontrol, School of Ecology, Shenzhen Campus of Sun Yat-sen University, Shenzhen 518107, China; State Key Laboratory of Biocontrol, School of Ecology, Shenzhen Campus of Sun Yat-sen University, Shenzhen 518107, China; State Key Laboratory for Vegetation Structure, Function and Construction (VegLab), MOE Key Laboratory of Biosystems Homeostasis & Protection and College of Life Sciences, Zhejiang University, Hangzhou 310058, China; State Key Laboratory of Biocontrol, School of Ecology, Shenzhen Campus of Sun Yat-sen University, Shenzhen 518107, China; CREAF, Cerdanyola del Vallès, Barcelona 08193 Catalonia, Spain; CSIC, Global Ecology Unit CREAF-CSIC-UAB, Bellaterra, Barcelona 08193 Catalonia, Spain; Département de Biologie, Université de Sherbrooke, Sherbrooke, Québec J1K 2R1, Canada; State Key Laboratory of Biocontrol, School of Ecology, Shenzhen Campus of Sun Yat-sen University, Shenzhen 518107, China

**Keywords:** ecosystem dynamics, plant functional traits, future projection, global mapping, machine learning, plant strategy

## Abstract

Understanding how terrestrial plant functional strategies (competitive, stress-tolerant, ruderal; CSR) respond to environmental conditions is crucial for predicting ecosystem dynamics under global climate change, yet remains unexplored at the global community level. Leveraging a machine learning approach, and utilizing multi-source satellite remote-sensing and field-collected sPlotOpen measurements data, we generated the first global community-level map of CSR functional strategy variations. Results show that S-selected strategies are globally dominant (C:S:R = 23.66:62.40:13.94%), with substantial spatial variations across biomes. This variability is strongly influenced by climatic variables (e.g. mean annual precipitation, diurnal temperature range) and soil properties (e.g. cation exchange capacity, total nitrogen). Future projections show that climate change favours S- (+0.33%) and R- (+0.31%) at the expense of C-selected strategy (−0.64%), alongside marked biome-specific shifts. Despite potential underestimation of localized climate uncertainties, these findings provide critical insights into global plant community dynamics under challenging abiotic conditions.

## INTRODUCTION

Plant strategies, the viable combinations of traits conferring fitness under varying selection pressures, reflect their evolutionary approach for survival and adaptation throughout their life history. These strategies also play a crucial role in shaping contemporary community assembly, vegetation succession, and overall ecosystem functioning [1,2]. Among the various frameworks developed for understanding these strategies, Grime’s competitive, stress-tolerant, ruderal (CSR) theory [3–5] stands out as a mature and prominent system, focusing on the balance of three core selection pressures: competition (the biotic limitations to biomass production from neighbours), stress (abiotic limitations to biomass accumulation), and disturbance (periodic destruction of biomass already produced). This framework coordinates plant species or individuals along a continuum defined by three primary strategies: competitive (C), stress-tolerant (S), and ruderal (R), with intermediate strategies also evident in this space. C strategies maximize resource acquisition and dominance in stable, productive environments with low disturbance; S strategies focus on maintaining cellular structures while regulating metabolic performance for survival in variable, unproductive environments; R strategies emphasize rapid lifecycles and regeneration in environments subject to frequent, intense disturbances [6]. Over the five decades since it was first proposed, CSR theory has provided a valuable framework for linking environment, vegetation functional traits, and ecosystem processes, especially carbon and nutrient cycling. Although its applicability at the species or individual level has been well-established across many local sites [7,8], its utility for understanding global community-level dynamics remains unclear. Specifically, how the interplay between climate, soil properties, and disturbance regimes shapes the global distribution of CSR strategies within plant communities, and how these strategies may shift under future climate change remain largely unsolved. Addressing these gaps is critical for predicting future ecosystem functioning and processes under global change [9,10].

At the species- or individual-level, Pierce *et al.* [8] have demonstrated that species among five of 14 major biomes (e.g. temperate grasslands, savannas, and shrublands) exhibited an averaged stress-tolerant ruderal and generalist (SR/CSR) strategy, while four (e.g. tropical and subtropical moist broadleaf forest) were characterized by a competitive ruderal and generalist (CS/CSR) strategy, others were dominated by intermediate strategies such as CSR, R/CSR, or CR/CSR, although considerable variations existed among species within the same biome. According to CSR theory, variation in the functional response of plants can be predicted or explained by differences in the intensity of stress and disturbance. Experimental and field studies have further demonstrated that S-selection was generally associated with external environment constraints (e.g. droughts or extreme climates), and R-selection was linked to the intensity of natural and anthropogenic disturbance events (e.g. fires, wind damage, and tillage) [11–13]. Stress and disturbance can, respectively, limit the rate of dry matter production or directly destroy plant biomass, and both therefore reduce ecosystem productivity. While these studies have advanced knowledge of CSR strategies across a wide range of vascular plant species globally, they provide limited understanding of how such strategies are manifested in communities. Community-level strategy composition could exhibit distinct patterns due to environmental or biotic filtering processes [14]. However, it is still unknown which strategy tends to predominate globally. How does variation in the expression of these strategies reflect the responses of plant communities to environmental conditions? What are the main drivers of spatial variation in plant strategies, and how do these strategies shift under climate change? Addressing these knowledge gaps is critical for predicting future ecosystem processes under global environmental change [9,10], and for enhancing the representation of complex plant communities in Earth System Models (ESMs) that inform global environmental policy and conservation [15].

Most community-level CSR strategies have remained limited to specific local sites, leaving biome-specific community-level CSR strategy signatures poorly understood due to the lack of global, continuous spatial-scale modelling. Some studies [16] assigned community CSR strategies via a practical tool, ‘StrateFy’ [8], using the community weighted mean (CWM) of key functional traits, e.g. specific leaf area (SLA), leaf dry matter content (LDMC), leaf area (LA). This method was constructed based on the trade-off between principal economics spectrum and size traits, and was calibrated using 3068 vascular plants from around the world. Other studies [17,18] directly modelled community CSR strategies by linking canopy hyperspectral reflectance to field-derived strategies using partial least squares regression. To advance global community-level CSR modelling, the potential solution is to integrate both principles by modelling the CWM traits, which can subsequently be used to calculate CSR strategies using the ‘StrateFy’ tool [8]. Multispectral remote sensing data (e.g. MODIS, Landsat, and Sentinel-2) combined with machine learning methods offer promising opportunities for global CWM leaf trait mapping [19,20]. However, global predictions of plant traits have generally focused on a few traits (e.g. SLA, LDMC), while LA was notably absent from many global mapping efforts. Furthermore, the calculation of CWM leaf traits in these studies relied on the spatial abundance of plant functional types (PFTs) derived from Landsat data (at a resolution of 30 m) rather than actual plant species composition. Meanwhile, most global models [20,21] integrated environmental variables, including soil and climatic factors, as predictors in statistical modelling, making it challenging to disentangle plant strategies from their environmental drivers when analysing strategy-environment relationships.

Here, we employed machine learning models using *in-situ* CWM trait data from the sPlotOpen database to provide a reference ‘ground truth’, alongside high-resolution satellite reflectance data, vegetation indices, vegetation seasonality, and topographical attributes as input features, to predict the three key plant economics/size traits of SLA, LDMC, and LA. The sPlotOpen database [22] is the largest open-access, spatially and environmentally balanced repository of CWM traits derived from *in-situ* trait observations and species composition in 95 104 plots, providing reliable ground truth data for global trait modelling. These traits were subsequently used to derive global community-level CSR strategy signature maps via the ‘StrateFy’ tool, which were then analysed to examine their relationships with environmental constraints associated with stress, disturbance, and productivity. Although CSR calculation relies on only three traits, these traits effectively capture the principal functional space of plant communities, strongly representing the trade-off between plant economics and size variation, and significantly correlate with a broader spectrum of plant characteristics, including whole-plant, leaf, stem, and seed traits [23,24] (see Methods). To further investigate potential shifts in plant strategies under future climate change, we employed random forest models to identify the drivers of CSR functional strategy variations and predict future dynamics. Given that most plants are predicted to encounter increasingly challenging future abiotic conditions, we hypothesized that stress-tolerance strategies will tend to predominate at the global scale and the geographic patterns of community-level variations would clearly respond to multiple environmental constraints, as predicted by CSR theory. We further expected that soil and climatic factors would drive these patterns, and that, under future climate scenarios, these strategies would shift towards greater stress-tolerance or ruderalism rather than competitor strategies.

## RESULTS AND DISCUSSION

### Advancing global mapping of community-level CSR strategy signatures

**Figure 1.**
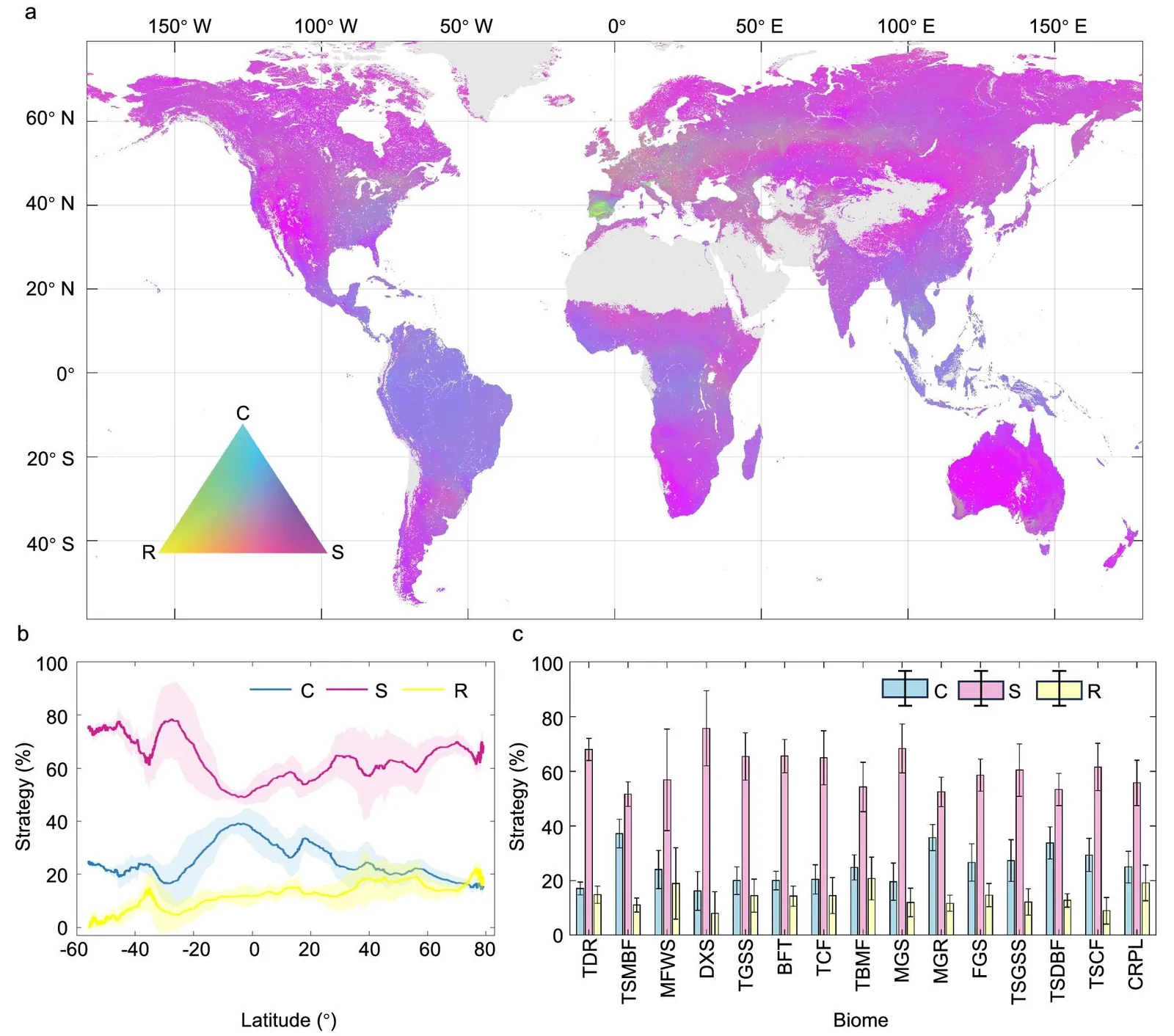
Global maps of community-level competitive, stress-tolerant, ruderal (CSR) strategy signatures. Panel (a) shows a combined representation of three plant strategies in a cyan-magenta-yellow (CMY) colour composite, with the degree of cyan, magenta, and yellow representing the extent of C-, S-, and R-selection, respectively. Panels (b) and (c) show the spatial distribution of CSR functional strategy signatures across latitudes and biomes, respectively. The shaded area in panel (b) indicates one standard deviation. The bar and error bar in panel (c) indicate the mean value and one standard deviation, respectively. TDR, tundra; TSMBF, tropical & subtropical moist broadleaf forests; MFWS, Mediterranean forests, woodlands & scrub; DXS, deserts & xeric shrublands; TGSS, temperate grasslands, savannas & shrublands; BFT, boreal forests/Taiga; TCF, temperate conifer forests; TBMF, temperate broadleaf & mixed forests; MGS, montane grasslands & shrublands; MGR, mangroves; FGS, flooded grasslands & savannas; TSGSS, tropical & subtropical grasslands, savannas & shrublands; TSDBF, tropical & subtropical dry broadleaf forests; TSCF, tropical & subtropical coniferous forests; CRPL, cropland. Review drawing number: GS 京(2026)1888号.

For the first time, we presented high-resolution (1×1 km) global maps of community-level CSR functional strategies (Fig. 1a and Fig. S1), revealing a global predominance of stress-tolerant (S) functional strategies, with competitive (C) and especially ruderal (R) strategies playing lesser roles (global averaged C:S:R = 23.66:62.40:13.94%). Using a more specific CSR classification scheme [8,25], 81.83% of plant communities showed a strong component of stress tolerance (S, S/CS, S/SR, and S/CSR; Fig. S2). This prevalence was further supported by sPlotOpen data, where 70.77% of plots were similarly classified (Fig. S3). Despite the overall global pattern, spatial heterogeneity was also evident. Europe was predominantly characterized by strategies tending towards the intermediate S/CSR class, Australia and western North America by the S class, Asia, eastern North America, southern South America, and northern and southern Africa by S/CS and S/CSR classes, and central Africa, South America, and southern Asia by CS/CSR and CS classes (Fig. S2). These results suggest that plants in most vegetation types undergo stress and mainly exhibit conservative resource economics adaptations, while the influence of disturbance events is not explicitly captured at a global scale. Although previous studies [26] have speculated this based on local observations or experiments which could be attributed to life history evolution under selection pressure, our findings provide the first quantitative evidence from both field-collected measurements and remote-sensing modelling at a global scale to support this hypothesis.

We analysed CSR strategy variation across latitudes (Fig. 1b) and biomes (Fig. 1c). Along the latitudinal gradient, the extent of S-selection exhibited a bimodal distribution, with higher prevalence in temperate regions (30°–70° and −20° to −40°) and lower prevalence in the tropics (−20° to 20°). In contrast, the extent of C-selection showed an inverse, complementary distribution to S (i.e. prevalent towards the tropics), while R-selection gradually increased with latitude. Given that three strategy extents are compositional and sum to 100%, an increase in one strategy necessarily corresponds to a decrease in at least one of the others. These patterns reflect plant functional adaptations to varying environmental conditions across latitudes. Tropical climates, being more stable, tend to support a higher prevalence of C-selected species that grow rapidly and dominate for long periods, while more unstable environments favour the S- and R- selected strategies due to the periodic nature of their challenges and relative instability [8]. These latitudinal patterns closely relate to that of species richness (Fig. S4), highlighting the distinct influences of environmental stress and disturbance on species richness [27,28]. Species richness tends to be low at both low levels of stress or disturbance due to competitive exclusion and high levels of stress or disturbance which limit survival for most species under severe conditions. Therefore, peak species richness occurs at intermediate levels of stress or disturbance (e.g. the tropics), where a balance between competitive exclusion and environmental filtering allows coexistence of diverse strategies, providing valuable insights for conservation and restoration efforts aimed at enhancing species richness.

When comparing across biomes (Fig. 1c), S-selection remained the most prevalent (S > 51.66%) and R-selection the least common (R < 20.82%), yet substantial variability persisted among biomes. More specifically, dense vegetation, such as tropical and subtropical moist and dry broadleaf forests and mangroves, exhibited relatively high extents of C-selection, but lower levels of S-selection, indicative of resource monopolization. In contrast, sparse vegetation, such as deserts and xeric shrublands, montane grasslands and shrublands, and tundra exhibited high prevalence of the S-selection, reflective of conservative resource use. Additionally, Mediterranean forests, woodlands and scrub, and temperate broadleaf and mixed forests, cropland exhibited a relatively high tendency towards R-selection, indicating adaptation to disturbance (i.e. rather than environmental factors that suppress metabolism; factors that outright kill cells and destroy biomass). Our findings align with prior biome-specific studies at the species level [8], which reported that grasslands exhibit higher S strategy values than forests and croplands exhibit higher R strategy values than natural vegetation. It also reported that tropical and subtropical moist broadleaf forests exhibited strategy convergence, with a distribution clustered around CS/CSR (43.00:42.00:15.00%), which closely aligned with our predicted CS/CSR (37.20:51.65:11.15%), while deserts or grasslands exhibited greater strategy variation, with a range from S-selected to R/CR-selected strategies. However, most previous research has primarily focused on individuals or species rather than communities. Our study uniquely reveals the distinct signatures of community-level CSR strategies across latitudes and biomes.

These distinctions reflect a manifestation of the resource economics spectrum at the community level, with plant strategy signatures spanning a continuum from resource conservation to resource acquisition [29]. Building upon the resource economics spectrum [30,31], our work presents a direct representation of plant functional strategies, offering two potential benefits. First, most trait-based approaches focus on resource investment strategies (acquisitive vs. conservative), but often overlook disturbance and environmental stress as explanatory factors, which play critical roles in community assembly and ecosystem functioning. The CSR strategy framework explicitly incorporates these aspects, which holds promise for offering additional predictive power for understanding vegetation responses to complex and changing environments. Second, growing evidence [32] suggests that species and community coexistence is not determined by individual traits alone, but by an optimized combination of traits that jointly confer fitness under selection. Thus, the CSR framework provides a complementary perspective to uncover mechanisms whereby certain trait combinations emerge under specific conditions.

### Linking plant functional strategies to environmental constraints

**Figure 2.**
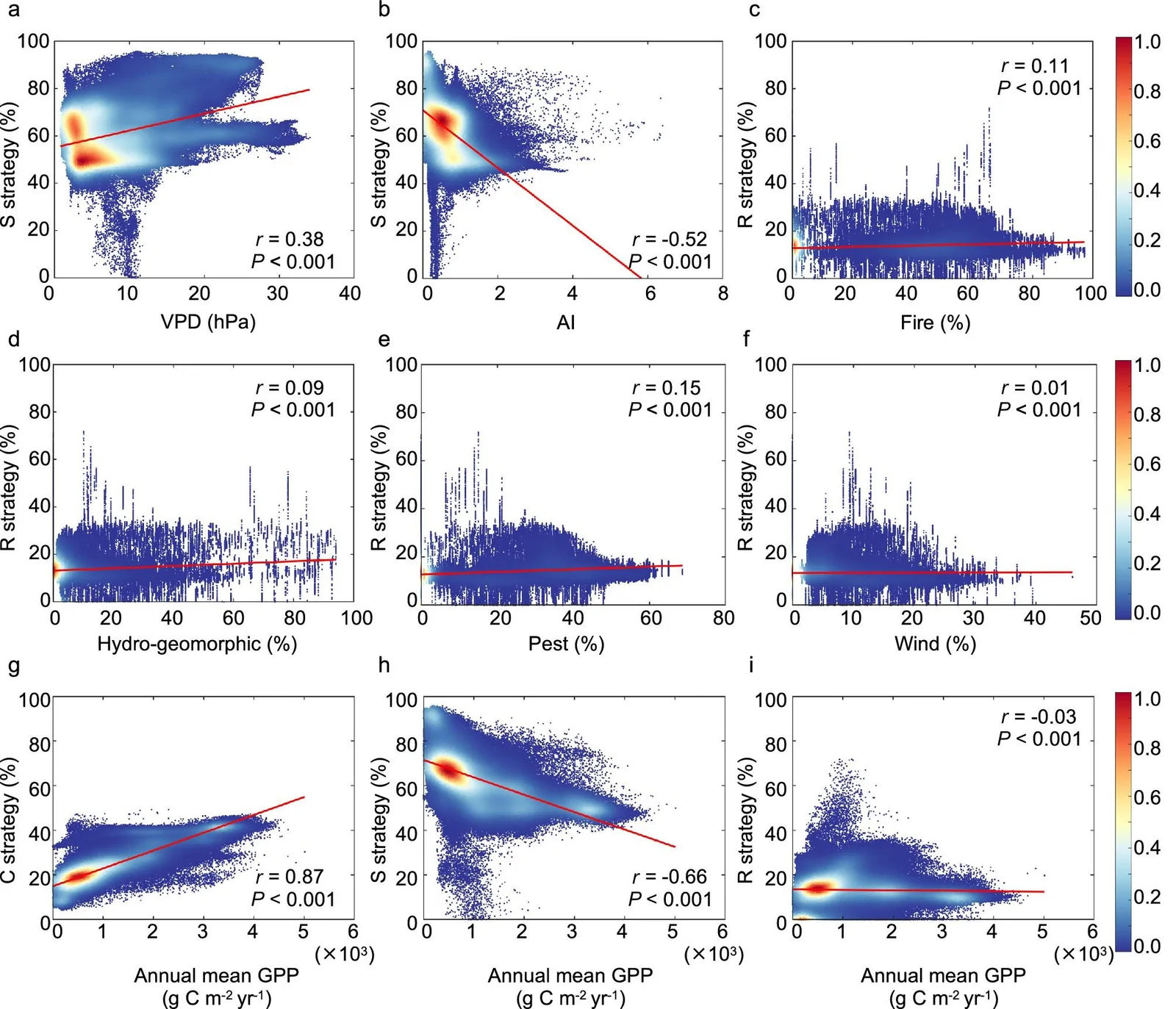
Density scatter plots showing the relationship between strategies and environmental constraints. S strategy and drought indices of vapor pressure deficit (VPD; a) and aridity index (AI; b); R strategy and four types of disturbance [fire (c), hydro-geomorphic (d), pest outbreak (e), and wind events (f)]; and C (g), S (h), and R (i) strategies and annual mean gross primary production (GPP). Red lines represent the linear regression fits between strategies and environmental constraints.

We cross-compared CSR strategy variation and environmental constraints at the global scale to examine how different ecological strategies correspond to stress, disturbance, and productivity-related environmental pressures. We first explored the relationship between the extent of S-selection and stress indicators, including two drought indices: vapor pressure deficit (VPD) and aridity index (AI). We also considered four temperature extreme indices, including TDcold and TDhot representing the frequency of extremely cold and hot days, and TNcold and TNhot denoting extremely cold and hot nights, all defined using 10th and 90th percentile thresholds. The results showed significant correlations across all indices, with Pearson’s *r* coefficients of 0.38 for VPD and −0.52 for AI (*P* < 0.001; Fig. 2a, b), and Pearson’s *r* of 0.24, −0.32, 0.33, −0.43 for TDcold, TDhot, TNcold, TNhot, respectively (*P* < 0.001; Fig. S5). These findings demonstrate that S-selection effectively represents vegetation responses to evident stress factors such as drought and temperature extremes. Next, we examined relationships between the extent of R-selection and both natural and anthropogenic disturbance. We observed consistent positive correlations for four major natural disturbance types (fire, hydro-geomorphic events, pest outbreaks, and wind events) across 782 global study sites (Pearson’s *r* = 0.11, 0.09, 0.15, and 0.01, respectively, *P* < 0.001; Fig. 2c–f) and two major anthropogenic disturbance types (artificial light at night, ALAN used as a proxy of urbanization and global cropping intensity for cropland expansion) across the globe (Pearson’s *r* = 0.22 and 0.28, respectively, *P* < 0.001; Fig. S6), supporting the interpretation of R-selection as a reliable indicator of disturbance-adapted vegetation. To assess how CSR strategies relate to ecosystem productivity, we used gross primary production (GPP) as a proxy for vegetation growth and ecosystem functioning. We found significant correlations between mean annual GPP and the extent of C-, S-, and R-selection, with Pearson’s *r* values of 0.87, −0.66, and −0.03, respectively (*P* < 0.001; Fig. 2g–i). These correlations were further confirmed using sPlotOpen plot data (Figs S7–S9).

These global cross-comparisons reveal distinct vegetation responses to varying environmental conditions, supporting core predictions of CSR theory. C strategists predominantly dominate in stable environments with fewer limiting environmental factors (i.e. stress; Fig. S10a, c), where they maximize photosynthesis and growth, thereby contributing to high ecosystem productivity (Fig. 2g). In contrast, S strategists prevail in environments characterized by harsh conditions such as drought (Fig. 2a, b) and temperature extremes (Fig. S5). These stressors often lead to reduced vegetative vigour and decreased productivity (Fig. 2h). R strategists, on the other hand, are primarily associated with environments experiencing frequent natural and anthropogenic disturbances at a global scale (Fig. 2c–f and Fig. S6) yet inducing relatively low stress (Fig. S10b, d), as the risks of mortality for individuals are offset by the regeneration of their populations following disruption, allowing them to persist while slightly reducing productivity in disturbed settings (Fig. 2i). To ensure comparability among strategies, we additionally evaluated all three CSR strategies using the same set of environmental variables (Fig. S11) and found that climate remained the dominant limiting factor overall. Notably, even for R-selection, climatic variables (e.g. VPD and AI) showed stronger associations than biotic factors, such as wind damage. The relatively stronger relationships observed for S- and C-selection suggest that these strategies are more directly constrained by the environmental variables considered, whereas R-selection may be influenced by additional local-scale or unaccounted-for factors (e.g. tree falls, local erosion). These broad-scale relationships between CSR strategies and environmental gradients likely arise because climatic conditions, disturbance regimes, productivity, and biotic interactions act as environmental filters, which select for certain traits and functional strategies suited to the prevailing set of conditions [33]. Incorporating experimental manipulations of specific environmental factors, such as water and nutrient availability, or disturbance regimes, are needed to directly test these filtering hypotheses. These findings align with previous smaller-scale *in-situ* studies [18,25,34], indicating that R strategists tend to grow fast but are short-lived in low-stress disturbed habitats, C-strategists are relatively long-lived in stable situations, while S strategists grow more slowly yet are extremely long-lived in limiting habitats. Taken together, these results provide additional evidence for the CSR theory at the plant community level, demonstrating its effectiveness in capturing the interplay between environmental conditions and ecosystem functioning. They also reinforce the utility of the CSR methodology as a robust tool for understanding plant-environment interactions across diverse ecological contexts globally.

### Future projections and shifts in plant functional strategies

**Figure 3.**
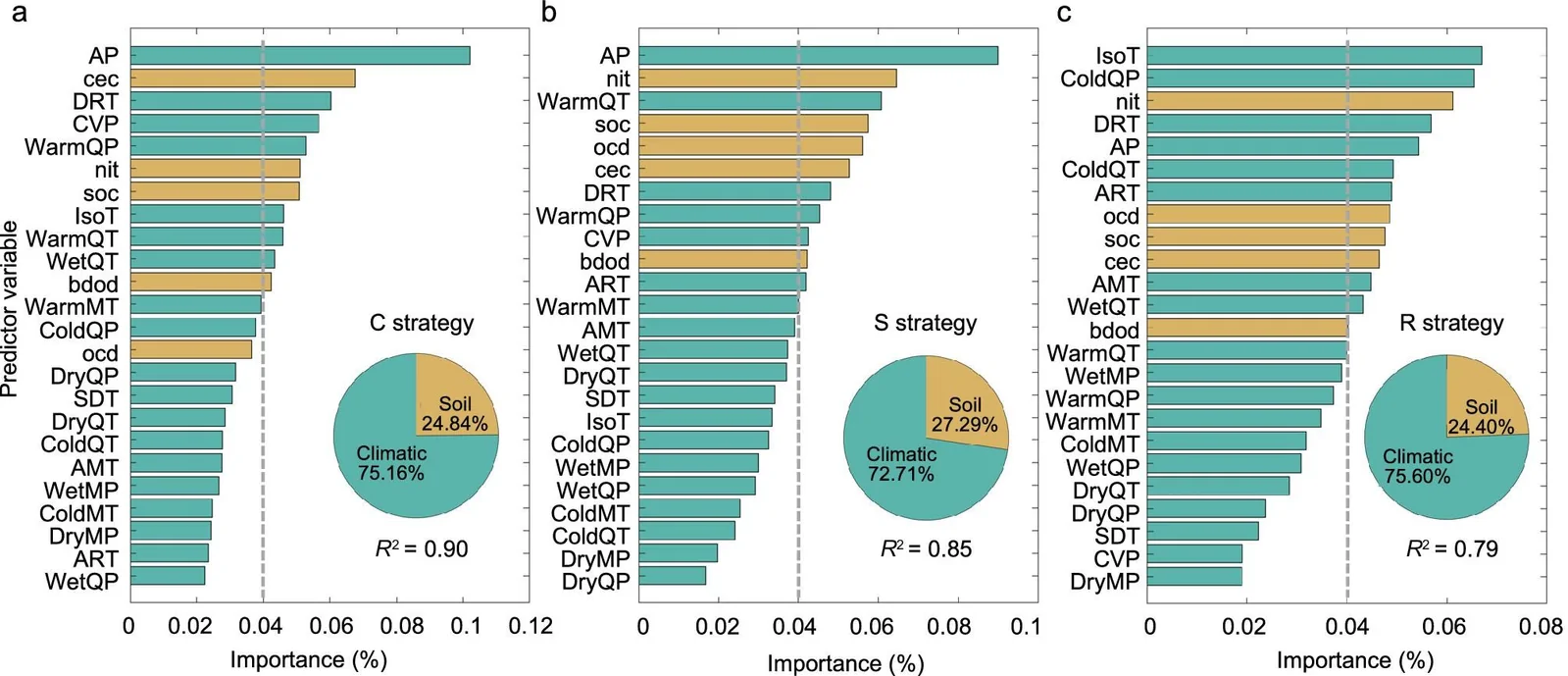
Relative importance (%) of climatic and soil variables in explaining variation in C (a), S (b), and R (c) strategies, as determined by random forest models. Climatic variables include AMT (annual mean temperature), DRT (mean diurnal range of temperature), IsoT (isothermality), SDT (temperature seasonality), WarmMT (max temperature of warmest month), ColdMT (min temperature of coldest month), ART (temperature annual range), WetQT (mean temperature of wettest quarter), DryQT (mean temperature of driest quarter), WarmQT (mean temperature of warmest quarter), ColdQT (mean temperature of coldest quarter), AP (annual precipitation), WetMP (precipitation of wettest month), DryMP (precipitation of driest month), CVP (precipitation seasonality), WetQP (precipitation of wettest quarter), DryQP (precipitation of driest quarter), WarmQP (precipitation of warmest quarter), ColdQP (precipitation of coldest quarter), and soil variables include bdod (bulk density), cec (cation exchange capacity), nit (total nitrogen), ocd (organic carbon density), soc (soil organic carbon content). Inset pie chart summarizes the relative contribution sourcing from climatic and soil variable groups.

Given the significant strategy-environment relationships, we used historical climatic data from WorldClim and soil properties from SoilGrids as input features in a random forest model to characterize CSR functional strategy signatures. These results highlighted the strong explanatory power of both climate and soil in shaping plant functional strategies, with *R*² values of 0.90, 0.85, and 0.79 (Fig. 3). Among the key predictors, mean diurnal range of temperature, annual mean precipitation, cation exchange capacity, and total nitrogen consistently showed high relative importance across all three strategies, with normalized importance values of 5.4-10.2%, 4.8%-6.0%, 4.6%-6.7% and 5.1%-6.5%, respectively. In addition to these overarching contributors, climate extremes further differentiated CSR functional strategies. For example, precipitation of the warmest and coldest quarters, mean temperature of the warmest quarter, and max temperature of the warmest month also played critical roles, illustrating that not only average conditions but also climate extremes impact photosynthesis and nutrient uptake. Particularly for C- and S-selection, precipitation seasonality (coefficient of variation) was a key factor, indicating that fluctuations in water availability influenced the resilience of stress-tolerant plants to withstand environmental constraints. Also for R-selection, isothermality played a crucial role, indicating that relatively stable environments may select R strategists to persist in habitats prone to recurring disturbances. Moreover, soil organic carbon content and organic carbon density were particularly important in explaining variation in S- and R-selection. Taken together, our results indicate that in addition to overall climate trends and soil properties, increasing climate variability and the rising frequency of extreme weather events are likely to have significant impacts on the distribution of CSR functional strategies, which in turn can influence the composition, diversity, and resilience of plant communities.

**Figure 4.**
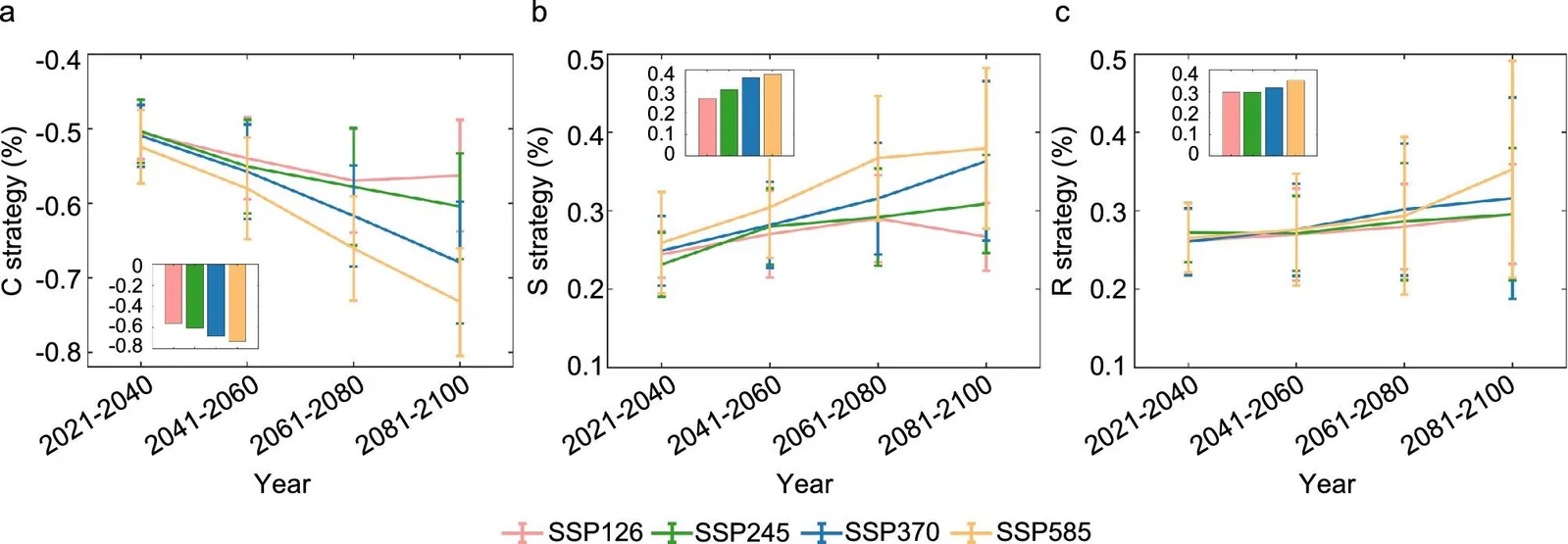
The average changes in C (a), S (b), and R (c) functional strategy signatures, from the historical period (1970–2000) to four future periods (2021–40, 2041–60, 2061–80, and 2081–2100). Future CSR signatures were predicted under CMIP6 future climate scenarios based on 10 global climate models (GCMs; see Materials) across four SSPs (126, 245, 370, and 585). The bar and error bar indicate the mean value and one standard deviation across the 10 climate models, respectively. The inset histogram shows the average changes in each variable from the current period to 2081–2100, where negative values indicate decreases and positive values indicate increases.

We next employed the derived random forest model to predict CSR functional strategy signatures under four CMIP6 future climate scenarios, Shared Socio-economic Pathway (SSP) 126, 246, 370, and 585, representing increasing levels of climate change severity relative to present conditions. From 2021 to 2100, although the global mean changes in C-, S-, and R-selection were relatively small, the spatial patterns revealed pronounced heterogeneity (Fig. 4a–c; Figs S12–S14). The extent of C-selection showed a consistent but modest global decline of 0.56% to 0.73%, with ~60% (59.65%–60.47%) of areas exhibiting negative changes. In contrast, S-selection showed a slight global increase by 0.27%–0.38%, yet positive changes occurred in about 54% (54.31%–54.75%) of areas, indicating substantial spatial variability. Similarly, R-selection increased moderately (0.30%–0.35%) at the global scale, with ~59% (59.10%–59.76%) of areas exhibiting positive changes. These results demonstrated that modest global averages masked considerable local differences across all SSPs. Across four future time intervals (2021–40, 2041–60, 2061–80, and 2081–2100), the model predicted a consistent decrease in C-selection, a rise in S-selection, and a slower but consistent increase in ruderalism. Across four SSPs, C-selection was predicted to decrease progressively from SSP126 to SSP585, S-selection to increase, with R-selection predicted to show the greatest increase under SSP585, followed by SSP370, SSP126, and SSP246. While the differences between specific SSP scenarios are obscured by high inter-model uncertainty in the extent of R-selection, the upward temporal trajectory is consistent across 70%–80% of the individual GCMs among four SSP scenarios (Fig. S15), indicating a robust directional response to climate change. This trend likely reflects the primary influence of climate and soil on CSR strategies, with soil properties themselves being partially shaped by climate. Thus, more severe climate change scenarios are expected to drive more pronounced shifts towards stress tolerance. Previous plot-level studies have suggested that climate change is likely to drive a shift towards stress-tolerant strategies [35,36] but without quantitative validation across large spatial and temporal scales. Our results here provide the first empirical support for these expectations, although further validation of these projections is still needed.

**Figure 5.**
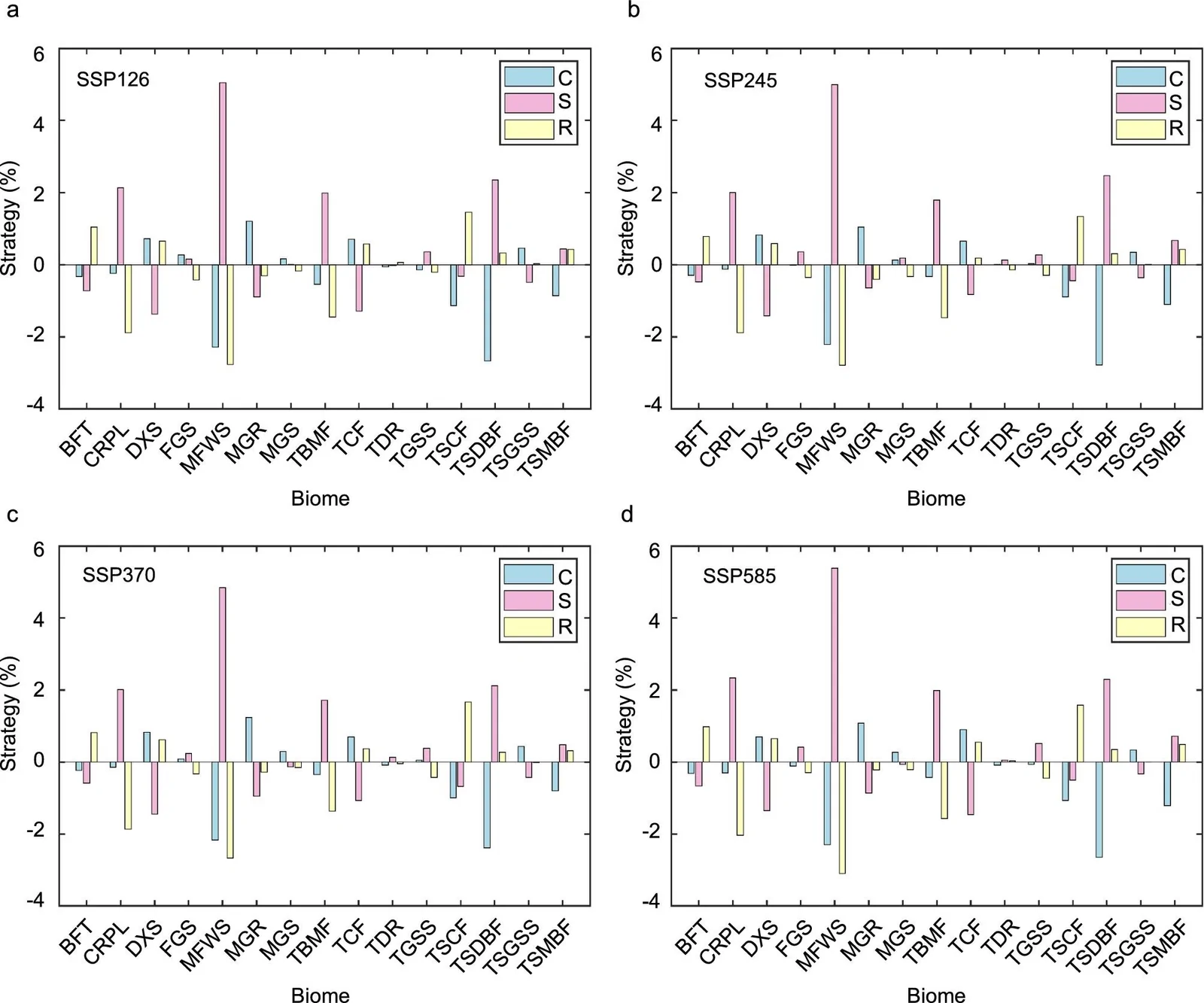
The average changes in CSR functional strategy signatures across biomes from the historical period (1970–2000) to 2100 under CMIP6 future climate scenarios, based on 10 global climate models (GCMs; see Materials) for SSP126 (a), SSP245 (b), SSP370 (c), and SSP585 (d). TDR, tundra; TSMBF, tropical & subtropical moist broadleaf forests; MFWS, Mediterranean forests, woodlands & scrub; DXS, deserts & xeric shrublands; TGSS, temperate grasslands, savannas & shrublands; BFT, boreal forests/Taiga; TCF, temperate conifer forests; TBMF, temperate broadleaf & mixed forests; MGS, montane grasslands & shrublands; MGR, mangroves; FGS, flooded grasslands & savannas; TSGSS, tropical & subtropical grasslands, savannas & shrublands; TSDBF, tropical & subtropical dry broadleaf forests; TSCF, tropical & subtropical coniferous forests; CRPL, cropland.

Across biomes, Mediterranean forests, woodlands, and scrub, temperate broadleaf and mixed forests, cropland, and tropical and subtropical dry broadleaf forests were predicted to exhibit the largest variations in CSR functional strategy signatures (>2%; Fig. 5). Specifically, these biomes were predicted to exhibit significant negative variations in CSR strategies, with either S or R strategies increasing or C strategies decreasing by more than 2%. Conversely, temperate conifer forests, mangroves, and deserts and xeric shrublands were predicted to display positive variations, with either S or R strategies decreasing or C strategies increasing by more than 1%. These projections align with local studies [37]. For example, in the Mediterranean area, which is identified as a climate change hotspot [38], plot-level research has reported an increase in stress-tolerant and a decline in ruderal strategies in herbaceous communities under climate and management change [37]. These shifts could occur through changes in species composition towards more conservative species. These biome-specific patterns suggest critical ecological processes such as succession and habitat shifts [17], highlighting the need for greater management and conservation focus on those ecosystems experiencing rapid strategy shifts under accelerating environmental pressures.

Projected shifts in plant functional strategies may be associated with decreases in GPP (Fig. S16). This reduction contrasts with the general increase in GPP projected by process-based models (e.g. DGVMs [39]), which is related to rising atmospheric CO_2_ concentrations [40]. Nevertheless, the upward trend is expected to slow down in the latter part of the century [41], highlighting the importance of accounting for processes that can suppress GPP growth (e.g. atmospheric dryness, nitrogen limitation, or competition). In this context, our results provide a complementary, trait-based perspective, suggesting that shifts towards more conservative plant strategies could potentially moderate productivity gains under changing environmental conditions. This underscores the need to enhance the representation of plant functional strategies and their influence on vegetation productivity in ESMs. Specifically, CSR dimensions could be incorporated as continuous trait axes to complement or refine existing PFTs-based schemes, either by mapping PFTs within a CSR space or by linking CSR values to key model parameters related to resource acquisition and stress tolerance. However, given the simplified, empirical nature of our prediction, these estimates should be interpreted cautiously and are not intended as robust projections of future GPP.

Our study represents a significant step towards understanding the global distribution of CSR strategies at the community level, but also highlights several limitations that warrant acknowledgement and identifies important avenues for future research. First, our analysis is based on spatially interpolated climate and soil data, which may not fully capture local-scale variations in environmental conditions that influence plant community composition. Future research should integrate higher-resolution data on microclimate, soil properties, and land management practices to refine the characterization of environmental drivers. Second, although our community-level strategy modelling is based on canopy reflectance data and visible canopy attributes often serve as proxies for less visible ecological phenomena due to the covariation of vegetation characteristics [17], this approach has limitations, particularly in landscapes with multiple canopy layers, such as dense forests. In such environments, the understorey vegetation or rarer (scarcely distributed) species play important roles in biodiversity maintenance but are often obscured, leading to potential inaccuracies. Moreover, our approach indicates adaptation strategies of plant communities to historical environmental conditions, but does not account for potential shifts in adaptation over time that would be reflected in changes of individuals/species functional traits in response to environmental changes and human intervention. This limitation arises from the lack of extensive and long-term field-based trait data. The use of new sensor technologies, such as those equipped on EnMap and HyspIRI satellites with higher spectral resolution, or SAR satellites with longer radar wavelengths could improve the accuracy of trait mapping and enable the characterization of three-dimensional vegetation structure. More importantly, artificial intelligence modelling algorithms effective with limited training samples, and worldwide collaborations among field researchers, citizen scientists, and other public and private research organizations, could improve our ability to capture fine-scale functional and structural changes over time. Integrating these advancements in future studies could enhance the characterization of plant functional strategies, especially in complex forested ecosystems and changing environments.

In summary, our study presents the first global map of community-level CSR strategy variations, serving as a powerful proxy for summarizing vegetation function worldwide and facilitating quantitative comparisons across contrasting environments and biomes [42]. With this unique global map, we found that stress-tolerant (S-selected) functional strategies dominate globally, highlighting that most plants are exposed to challenging abiotic conditions. Consistent with the predictions of CSR theory, the distribution of strategies reflects community responses to environmental conditions worldwide, which is crucial for predicting community dynamics under global climate change. Lastly, both climate and soil emerge as key drivers of spatial variations in CSR strategies, and future projections indicate shifts towards stress-tolerant and ruderal strategies, highlighting the increasingly harsh environments that ecosystems are facing. These findings represent a major advance, building upon Grime’s scientific legacy, and underscore the value of community-level CSR strategy as a direct and predictive tool to link biotic responses of plant communities to ecosystem functions, while offering insights into key processes, such as resistance and resilience to disturbances, and successional dynamics [43–45]. They also highlight the urgent need to monitor the shifts in plant strategies under increasing selection pressures from disturbance and stress, which are critical for improving projections in ESMs and guiding future conservation and management efforts.

## MATERIALS AND METHODS

### Data sources

Ground-based functional traits were derived from the sPlotOpen dataset (*n* = 95 104 plots), the largest globally distributed vegetation plot database. We focused on three community-weighted mean (CWM) traits, SLA, LDMC, and leaf area (LA), to characterize CSR strategies. Outliers (>5 standard deviations within PFTs) were removed, yielding 92 848 observations.

Remote sensing observations were obtained primarily from MODIS products. We used BRDF-adjusted reflectance (MCD43A4, 500 m, 2000–23) to derive vegetation indices (EVI, NDVI, MNDWI, BSI) and seasonal metrics (NDVI phase and amplitude via harmonic analysis). Land cover (MOD12Q1), habitat heterogeneity (contrast and evenness from EVI texture), and topographic variables (elevation, slope, aspect from ASTER GDEM) were also included.

Climatic variables (19 bioclimatic variables, ~1 km) were sourced from WorldClim. Historical data (1970–2000) were used for modelling, while future projections (CMIP6; 10 GCMs; SSP126, 245, 370, 585; four time periods from 2021 to 2100) supported scenario analysis. Soil properties were obtained from SoilGrids (250 m resolution). Five key variables (soil organic carbon, bulk density, cation exchange capacity, nitrogen, and organic carbon density) were aggregated across depths.

Environmental constraints included stress (VPD, AI, and temperature extremes), disturbance (natural: fire, hydro-geomorphic, pests, wind; anthropogenic: ALAN and cropping intensity), and productivity (mean annual GPP from GOSIF, 2000–23). All large-scale data processing was conducted on Google Earth Engine.

### Trait modelling and CSR mapping

We modelled global distributions of SLA, LDMC, and LA at 1 km resolution using random forest regression. A total of 19 predictors were used, including reflectance bands, vegetation indices, seasonality metrics, land cover, habitat heterogeneity, and topography. Predictors were temporally averaged and also aggregated to a coarser scale (10 000 km) to account for spatial autocorrelation.

Model performance was evaluated using five-fold cross-validation, with normalized RMSE and *R*^2^ as metrics. Final models minimized prediction error and maximized explanatory power. Variable importance was assessed using mean decrease in impurity and standardized (0–1).

Predicted trait maps were translated into CSR strategy signatures using the StrateFy framework, which quantifies competitive (C), stress-tolerant (S), and ruderal (R) strategies based on SLA, LDMC, and LA. To validate this trait space, we conducted a PCA on 18 CWM traits, confirming that size and economics axes explained 51.5% of variance and aligned with CSR dimensions. Model accuracy was high (*n*RMSE = 0.05–0.11; *R*^2^ = 0.54–0.62), and derived CSR strategies showed good agreement with in situ estimates (*n*RMSE = 0.10–0.16; *R*^2^ = 0.41–0.42).

### Environmental relationships and projections

We assessed whether CSR strategies reflect ecological niches by examining correlations with environmental constraints. Specifically, S strategies were related to drought and temperature extremes; R strategies to natural and anthropogenic disturbances; and all strategies to productivity (GPP). Pearson correlations and linear regressions were applied.

Future shifts in CSR strategies were projected using a random forest model trained on historical climate and soil data, then applied to CMIP6 projections across multiple GCMs, SSPs, and time periods (2021–2100). More detailed information can be found in the Supplementary Materials.

## Supplementary Material

nwag416_Supplemental_File

## Data Availability

All data and materials used in the analysis are publicly accessible. The code was made available in GitHub (https://github.com/jing-sysu/CSRstrategy) for review. The CSR strategy maps were uploaded in Google Earth Engine (GEE): https://code.earthengine.google.com/?asset=projects/storied-smile-439400-s6/assets/C_stra; https://code.earthengine.google.com/?asset=projects/storied-smile-439400-s6/assets/S_stra; https://code.earthengine.google.com/?asset=projects/storied-smile-439400-s6/assets/R_stra.
