# Supplementary material for: Facing a harsh climate: terrestrial plant functional strategies in a changing world
Source: Natl Sci Rev. 2026 Jul 14;13(16):nwag416. doi: 10.1093/nsr/nwag416 (PMC13525714; doi:10.1093/nsr/nwag416)
Supplement: Supplementary file 1 [file nwag416_supplemental_file.docx]

**Supplementary Information**

**Title:** Facing a harsh climate: Terrestrial plant functional strategies in a changing world

**Author List:** Jing Wang*, Simon Pierce, Yuanzhi Li, Donghao Wu, Heng Huang, Josep Peñuelas, Bill Shipley, Chengjin Chu*

**Corresponding Authors:** chuchjin@mail.sysu.edu.cn; wangj895@mail.sysu.edu.cn

**Materials and Methods**

**Supplementary Figures:** 22 figures

**Figure S1** Global maps of the three plant strategies, competitive (C; a), stress-tolerant (S; b), and ruderal (R; c).

**Figure S2** Global map of tertiary CSR plant strategies.

**Figure S3** Global maps of community-level CSR strategies derived from *in-situ* sPlotOpen trait measurements (*n*=92,848).

**Figure S4** Comparison of latitudinal patterns between plant alpha diversity and CSR functional strategy signatures.

**Figure S5** Density scatter plots showing the relationship between predicted S strategy and four temperature extreme indices, TDcold (a), TDhot (b), TNcold (c), and TNhot (d).

**Figure S6** Density scatter plots showing the relationship between predicted R strategy and two anthropogenic disturbance indices, artificial light at night (ALAN; a) and global cropping intensity (GCI; b).

**Figure S7** Density scatter plots showing the relationship between actual strategies and environmental constraints.

**Figure S8** Density scatter plots showing the relationship between actual S strategy and four temperature extreme indices, TDcold (a), TDhot (b), TNcold (c), and TNhot (d).

**Figure S9** Density scatter plots showing the relationship between actual R strategy and two anthropogenic disturbance indices, ALAN (a) and GCI (b).

**Figure S10** Density scatter plots showing the relationship between drought indices and two strategies, VPD vs. C (a) and R (b); and AI vs. C (c) and R (d).

**Figure S11.** Pearson correlation matrix for the key biotic and abiotic factors and CSR strategies.

**Figure S12** The predicted changes in C functional strategy signatures from the historical period (1970-2000) to 2100.

**Figure S13** The predicted changes in S functional strategy signatures from the historical period (1970-2000) to 2100.

**Figure S14** The predicted changes in R functional strategy signatures from the historical period (1970-2000) to 2100.

**Figure S15** Predicted average changes in R functional strategy signatures across four future periods based on 10 global climate models (GCMs) under CMIP6 future climate scenario.

**Figure S16** The average changes in GPP from the historical period (1970-2000) to four future periods (2021-2040, 2041-2060, 2061-2080, and 2081-2100).

**Figure S17** Flowchart summarizing the materials and methods.

**Figure S18** Principal component analyses (PCA) of eighteen community weighted mean (CWM) traits from sPlotOpen dataset (*n* = 95,104).

**Figure S19** Global maps of three functional traits predicted using random forest models: specific leaf area (SLA, a), leaf dry matter content (LDMC, b), and leaf area (LA, c).

**Figure S20** Scatter plots comparing predicted and *in-situ* trait measurements from the testing dataset for SLA (a), LDMC (b), and LA (c).

**Figure S21** Comparison of trait mapping for LDMC and SLA across seven plant functional types between the approach of Moreno-Martínez, et al. ^1^ and our method.

**Figure S22** Density scatter plots comparing predicted and actual strategies derived from *in-situ* sPlotOpen trait measurements for C (a), S (b), and R (c) strategies.

**Materials**

Four kinds of data were used in this study, including ground-based functional trait observations, remote sensing observations, climatic and soil data, and environmental data associated with stress, disturbance, and productivity as shown below. For clarity, a flowchart summarizing the materials and methods is presented in Fig. S17.

**Functional traits**

To build a global model for predicting functional traits, we used the sPlotOpen dataset as ground truth^2^. sPlotOpen is the largest open-access dataset of vegetation plots ever released (*n* = 95,104), which is an environmentally and spatially balanced subset of the global sPlot vegetation plots dataset v.2.1^3^. The dataset included 18 community weighted mean (CWM) traits sourced from the gap-filled version of the TRY database, spanning a period from 1888 to 2015^4,5^. For this study, we focused on three key CWM traits, specific leaf area (SLA), leaf dry matter content (LDMC), and leaf area (LA), to model CSR functional strategy signatures. The sPlotOpen data was accessed via the iDiv data repository (<https://idata.idiv.de>). To minimize noise from data compilation (e.g., gap-filling), we removed outliers defined as observations exceeding five standard deviations from the mean trait value within a given plant function type (e.g., forest, shrub, grass), resulting trait measurements of 92,848.

**Remote sensing observations**

- **MODIS reflectance, vegetation indices, and landcover type**

To derive input features for global functional trait modelling, we used the MODIS BRDF-adjusted reflectance product, MCD43A4, and the MODIS land cover product, MOD12Q1^6^, both at a spatial resolution of 500 m. MCD43A4 data acquired between 2000 and 2023 were used to obtain monthly gap-free reflectance estimates by means of the quality assessment (QA) information stored as a separate quality product, MCD43A2^7^. From MCD43A4 data, we investigated four vegetation indices, the enhanced vegetation index (EVI)^8^, the normalized difference vegetation index (NDVI)^9^, the modified normalized difference water index (MNDWI)^10^, and bare soil index (BSI)^11^. To estimate vegetation seasonality, we analysed the phase and amplitude of NDVI time series using a harmonic modelling approach.

- **Habitat heterogeneity data**

Additionally, we used global habitat heterogeneity data, which quantifies spatial heterogeneity of global habitat at a 1 km resolution based on the textural features of the MODIS EVI from 2001 to 2005^12^. Among the 14 available metrics, we selected contrast and evenness as key input features for global functional trait modelling.

- **Topography data**

We also used the Advanced Spaceborne Thermal Emission and Reflection Radiometer (ASTER) Global Digital Elevation Map (GDEM) Version 3^13^, a 30 m spatial resolution DEM dataset, for global functional trait modelling. This dataset offers a great advantage in coverage, spanning from 83°N to 83°S and encompassing 99% of Earth’s landmass. From this elevation data, we derived two other topographic attributes, aspect and slope.

**Climatic** **and soil data**

- **Climatic data**

We used the WorldClim climatic data^14^ at ~1 km (30 arc-second) spatial resolution. The dataset includes 19 bioclimatic variables, which are derived from the monthly temperature and rainfall data, in order to represent annual trends (e.g., mean annual temperature, annual precipitation), seasonality (e.g., annual range in temperature and precipitation), and extreme or limiting factors (e.g., temperature of the coldest and warmest month, and precipitation of the wet and dry quarters). This dataset includes both historical (1970-2000) and future climate data (2021-2100) under Phase Six of the Coupled Model Intercomparison Project (CMIP6) projections. The future climate data were processed for 23 global climate models (GCMs) and four Shared Socio-economic Pathways (SSPs): 126, 245, 370, and 585. In this study, historical climate data were used for modelling, while future climate projections from 10 models (ACCESS-CM2, BCC-CSM2-MR, CMCC-ESM2, EC-Earth3-Veg, INM-CM5-0, IPSL-CM6A-LR, MIROC6, MPI-ESM1-2-HR, MRI-ESM2-0, and UKESM1-0-LL) covering four time periods (2021-2040, 2041-2060, 2061-2080, and 2081-2100) were applied for predictions.

- **Soil data**

We used SoilGrids version 2.0, a globally-compiled dataset of soil properties at a spatial resolution of 250 meters^15^. The dataset includes 11 soil properties: pH, soil organic carbon content (soc), bulk density (bdod), cation exchange capacity (cec), total nitrogen (nit), soil organic carbon density (ocd), soil organic carbon stock, coarse fragments content, sand content, silt content, and clay content, measured at six standard depths (up to 200 cm). These data were derived using globally fitted models, based on approximately 240,000 soil profile observations worldwide and a series of environmental covariates, describing vegetation, terrain morphology, climate, geology, and hydrology. And the robustness and effectiveness of this dataset have been quantitatively evaluated across global, continental, and regional scales. In this study, we used five nutrient properties, soc, bdod, cec, nit, and ocd, and calculated the sum of these soil properties across all depths for modelling.

**Environmental data associated with stress, disturbance, and productivity**

- **Stress data**

To quantify environmental constraints associated with stress, we used two drought indices, vapor pressure deficit (VPD) and aridity index (AI), and four temperature extreme indices, TDcold, TDhot, TNcold, and TNhot, at a spatial resolution of 0.5°. VPD and AI datasets evaluate drought conditions relying on factors such as precipitation, evapotranspiration, temperature, and relative humidity. They can be accessed from the European Centre for Medium-Range Weather Forecasts (ECMWF) ERA5 Land (https://cds.climate.copernicus.eu/datasets/reanalysis-era5-land) and Consortium of Spatial Information (CGIAR-CSI) website (www.cgiar-csi.org). The TDcold, TDhot, TNcold, and TNhot datasets evaluate extreme temperature events from 1951 to 2014 using the NorESM2-LM climate model (Norwegian Earth System Model version 2, Low-Resolution). These datasets quantify the frequency of extreme cold and hot days (TDcold, TDhot) and nights (TNcold, TNhot) using 10th and 90th percentile thresholds over a 5-day moving window during a baseline period between 1961 and 1990.

- **Disturbance data**

To quantify environmental constraints associated with both natural and anthropogenic disturbance, we used a dataset for assessing natural disturbance which was derived from a meta-analysis that documents four types of disturbance: fire, hydro-geomorphic, pest outbreak, and wind events spanning 1990 to 2020 from 782 global study sites^16^ and two datasets for assessing anthropogenic disturbance, which include the averaged 1km artificial light at night (ALAN) dataset^17^ from 1992 to 2021 and the averaged 250-m global cropping intensity (GCI)^18^ from 2001 to 2019.

- **Gross primary production (GPP) product**

To quantify environmental constraints associated with productivity, we used GOSIF GPP dataset, a global gross primary production product at a spatial resolution of 0.05°^19^. This product maps GPP using linear relationships between SIF (that is derived from the global, OCO-2-based SIF product; GOSIF) and GPP. This product has been evaluated at both eddy covariance flux sites and grid cell levels. We calculated the mean annual GPP from 2000 to 2023 to indicate the averaged ecosystem productivity.

Due to the global scope of this study along with the significant computational and storage demands associated with the above datasets, we used the Google Earth Engine (GEE) platform for remote sensing data accessing, processing, and analysis^20^.

**Methods**

**Mapping global community-level CSR strategy** **signatures**

We modelled the global CWM of each trait (SLA, LDMC, and LA) at the pixel level (1 🞩 1 km) using a random forest (RF) regression algorithm^21^ on the GEE platform. RF regression is a non-parametric machine learning algorithm known for its robustness to overfitting and its ability to handle variable inputs due to the bagging process and random feature selection^22^. It has been widely used to model and predict ecological and remote sensing data within and across ecosystems^23^.

In this study, we gathered a total of 19 variables, including median reflectance of MODIS bands (band 1 to 7; *n*=7), vegetation indices (EVI, NDVI, MNDWI, BSI; *n*=4), vegetation seasonality (phase and amplitude of NDVI time series; *n*=2), land cover type (*n*=1), habitat heterogeneity metrics (contrast and evenness; *n*=2), and topographical attributes (elevation, slope, aspect; *n*=3). To minimize temporal variability and uncertainty, we averaged these variables across the available time periods. These variables were used both at a spatial resolution of 1 km and at an upscaled resolution of 10,000 km as input features for global functional trait modelling. The upscaled resolution used here is to consider spatial autocorrelation. The RF regression model was trained and evaluated using a commonly-used five-fold cross-validation method. In this method, ground-truth samples were randomly split into five groups of equal size, with any of the four groups used for model training and the remaining group for model testing. This process was iterated until every possible combination of groups had been used for evaluation. The normalized root mean squared error (*n*RMSE; precision) and R square (*R*^2^; goodness of fit) were calculated for the model performance evaluation. The final model was optimized to minimize cross-validation errors, with the lowest *n*RMSE and highest *R*^2^, and was subsequently used to map the three traits globally. Variable importance was determined by calculating the decrease in node impurities from splitting on each variable, averaged over all trees, and derived from the Out of Bag (OOB) error. These values were then standardized to a 0-1 scale for comparison.

Once the global trait maps were derived, CSR strategies for each pixel were assigned using the globally applicable “StrateFy” framework provided by Pierce, et al. ^24^. This method incorporates economics spectrum (SLA, with high values representing acquisitive economics, and LDMC, with high values representing conservative economics) and size (LA) traits to calculate CSR strategy scores (%) and assign strategy classes, using a spreadsheet tool calibrated on 3068 vascular plants. This method has been extensively validated across diverse global sites^25^ and controlled experimental settings^26,27^, providing a reliable framework for quantitatively comparing the plant strategies across highly contrasting habitats. To further validate the effectiveness of this method, we conducted a principal component analysis (PCA) on 18 CWM traits from sPlotOpen dataset (*n* = 95,104). The first two principal components (PCs) together explained 51.51% of total trait variance (Fig. S18). PC1 (31.99% of variance) was correlated most strongly with plant size dimensions such as seed mass and leaf area (*r*=0.89 and 0.76, *P*<0.001), while PC2 (19.52% of variance) was correlated most strongly with economics spectrum dimensions such as SLA and LDMC (*r*= 0.92 and -0.77, *P*<0.001). Consistent with previous studies^28,29^, the results again demonstrate that the fundamental trait trade-offs underpin plant adaptive specialization. Although only three traits are used for CSR calculation, our study (Fig. S18) confirms that they effectively capture the principle functional space of plant adaptation and are significantly correlated with a broader spectrum of plant characteristics, including whole-plant traits (e.g., plant height), leaf traits (e.g., leaf nitrogen and carbon concentrations), stem traits (e.g., stem specific density), and seed traits (e.g., seed mass and length), which is consistent with Pierce, et al. ^24^ but with a larger sample size across traits.

We evaluated the spatial modelling performance for three CWM traits, SLA, LDMC, and LA (Fig. S19), using the testing dataset. The trait modelling evaluation shows that the random forest regression models yielded consistently high accuracies for all three traits, with normalized RMSE (*n*RMSE) values of 0.10, 0.11, and 0.05 and *R*² values of 0.57, 0.54, and 0.62 (*P*<0.001), respectively (Fig. S20). The model predictions demonstrated good alignment with both the best linear fit regression and the one-to-one line, establishing a reliable benchmark for analyzing CSR strategies. Although cross-comparisons with the SLA and LDMC results from a previous modelling study^1^ show slight differences in magnitude (potentially due to differences in the ground truth datasets used), the findings generally align well across different plant functional types (Fig. S21). We further assessed the accuracy of the predicted CSR functional strategy signatures derived from the validated traits against those derived from *in-situ* trait measurements in the sPlotOpen dataset (Fig. S22). The results demonstrated high accuracy, with a *n*RMSE of 0.10-0.16 and an *R*^2^ value of 0.41-0.42 across all strategies. These results taken together suggest we have established a reliable benchmark for analysing community-level CSR strategy signatures.

**Exploring the relationships between CSR functional strategy signatures and environmental constraints**

To evaluate whether the derived CSR functional strategy signatures represent ecological niches, we examined their relationships with environmental constraints associated with stress, disturbance, and productivity. Specifically, we first analysed the relationship between S strategy and two drought indices (VPD and AI) and four temperature extreme indices (TDcold, TDhot, TNcold, TNhot). Second, we investigated the relationship between R strategy and four natural disturbance types (fire, hydro-geomorphic, pest outbreak, and wind events) and two anthropogenic disturbance types (urbanization and cropland expansion). Third, we assessed the relationship between CSR functional strategy signatures and mean annual GPP. All analyses used Pearson’s correlation coefficient (*r*) and significance level (*P*) derived from least-squares linear regression.

**Future projection of CSR strategy** **signatures and GPP**

To predict shifts in CSR functional strategy signatures under future climate scenarios, we employed a random forest model using historical WorldClim climatic data and SoilGrids soil data as input features. Due to the large volume of data and computational requirements, all datasets were consistently resampled to a spatial resolution of 5 km prior to analysis. The derived model was then applied to CMIP6 future climate projections from 10 global climate models (GCMs) across four SSPs (126, 245, 370, and 585) and four future time periods: 2021-2040, 2041-2060, 2061-2080, and 2081-2100. Future GPP was estimated indirectly by first projecting shifts in CSR strategy dominance under future environmental conditions, and then applying an empirical relationship between CSR strategies and GPP derived from present-day observations.

**Figure S1** Global maps of the three plant strategies, competitive (C; a), stress-tolerant (S; b), and ruderal (R; c).

**Figure S2** Global map of tertiary CSR plant strategies, categorized using the classification developed by Hodgson, et al. ^30^ and Pierce, et al. ^24^. The inset figure shows the histogram distribution of different strategy classes (*n*=19).

**Figure S3** Global maps of community-level CSR strategies derived from *in-situ* sPlotOpen trait measurements (*n*=92,848). Panel (a) shows a combined representation of three plant strategies in a cyan-magenta-yellow (CMY) colour composite, with the degree of cyan, magenta, and yellow representing the extent of C-, S-, and R-selection, respectively. Panel (b) shows the global class map of plant strategies, which was categorized using the methods described by Hodgson, et al. ^30^ and Pierce, et al. ^24^. The inset figure shows the histogram distribution of different strategy classes (*n*=19).

**Figure S4** Comparison of latitudinal patterns between plant alpha diversity and CSR functional strategy signatures. Alpha diversity data were derived from Sabatini, et al. ^31^.

**Figure S5** Density scatter plots showing the relationship between predicted S strategy and four temperature extreme indices, TDcold (a), TDhot (b), TNcold (c), and TNhot (d). The TDcold, TDhot, TNcold, and TNhot datasets evaluate extreme temperature events based on the frequency of extreme cold and hot days (TDcold, TDhot) and nights (TNcold, TNhot) using 10th and 90th percentile thresholds over a 5-day moving window during a base period. Red lines represent the linear regression fits between S strategy and temperature extreme indices.

**Figure S6** Density scatter plots showing the relationship between predicted R strategy and two anthropogenic disturbance indices, artificial light at night (ALAN; a) and global cropping intensity (GCI; b). Red lines represent the linear regression fits between R strategy and disturbance indices.

**Figure S7** Density scatter plots showing the relationship between actual strategies and environmental constraints. S strategy with drought indices of vapor pressure deficit (VPD; a) and aridity index (AI; b); actual R strategy with four types of disturbance (fire (c), hydro-geomorphic (d), pest outbreak (e), and wind events (f)); and actual C (g), S (h), and R (i) strategies with mean annual gross primary production (GPP). Actual CSR strategies were derived from *in-situ* sPlotOpen trait measurements (*n*=92,848; Fig. S3). Red lines represent the linear regression fits between strategies and environmental constraints.

**Figure S8** Density scatter plots showing the relationship between actual S strategy and four temperature extreme indices, TDcold (a), TDhot (b), TNcold (c), and TNhot (d). Actual CSR strategies were derived from *in-situ* sPlotOpen trait measurements (*n*=92,848; Fig. S3). The TDcold, TDhot, TNcold, and TNhot datasets evaluate extreme temperature events based on the frequency of extreme cold and hot days (TDcold, TDhot) and nights (TNcold, TNhot) using 10th and 90th percentile thresholds over a 5-day moving window during a base period. Red lines represent the linear regression fits between S strategy and temperature extreme indices.

**Figure S9** Density scatter plots showing the relationship between actual R strategy and two anthropogenic disturbance indices, ALAN (a) and GCI (b). Actual R strategy was derived from *in-situ* sPlotOpen trait measurements (*n*=92,848; Fig. S3). Red lines represent the linear regression fits between R strategy and disturbance indices.

**Figure S10** Density scatter plots showing the relationship between drought indices and two strategies, VPD vs. C (a) and R (b); and AI vs. C (c) and R (d). Red lines represent the linear regression fits between the strategies and drought indices.

**Figure S11.** Pearson correlation matrix for the key biotic and abiotic factors and CSR strategies. The markers “ *** ”, “ ** ”, “ * ” indicate that the correlation is significant at the 0.001, 0.01, and 0.05 probability levels, respectively.

**Figure S12** The predicted changes in C functional strategy signatures from the historical period (1970-2000) to 2100, represented by the mean and standard deviation across 10 global climate models (GCMs) under CMIP6 future climate scenarios, SSP126, SSP245, SSP370, and SSP585. The inset histogram shows the frequency distribution of average changes and standard deviations in the C signatures from the historical period to 2100.

**Figure S13** The predicted changes in S functional strategy signatures from the historical period (1970-2000) to 2100, represented by the mean and standard deviation across 10 global climate models (GCMs) under CMIP6 future climate scenarios, SSP126, SSP245, SSP370, and SSP585. The inset histogram shows the frequency distribution of average changes and standard deviations in the S signatures from the historical period to 2100.

**Figure S14** The predicted changes in R functional strategy signatures from the historical period (1970-2000) to 2100, represented by the mean and standard deviation across 10 global climate models (GCMs) under CMIP6 future climate scenarios, SSP126, SSP245, SSP370, and SSP585. The inset histogram shows the frequency distribution of average changes and standard deviations in the R signatures from the historical period to 2100.

**Figure S15** Predicted average changes in R functional strategy signatures across four future periods (2021-2040, 2041-2060, 2061-2080, and 2081-2100) based on 10 global climate models (GCMs; see Materials and Methods) under CMIP6 future climate scenarios SSP126 (a), SSP245 (b), SSP370 (c), and SSP585 (d). An upward trend from 2021 to 2100 was projected by 8 of 10 GCMs for SSP126, and by 7 of 10 GCMs for SSP245, SSP370, and SSP585.

**Fig. S16** The average changes in GPP from the historical period (1970-2000) to four future periods (2021-2040, 2041-2060, 2061-2080, and 2081-2100). The bar and error bar indicate the mean value and one standard deviation across the 10 climate models, respectively. The inset histogram shows the average changes in GPP from the current period to 2081-2100, where negative values indicate decreases and positive values indicate increases.

**Figure S17** Flowchart summarizing the materials and methods: 1) mapping three key CWM traits (SLA, LDMC, and LA) using random forest regression models with *in-situ* CWM trait data from the sPlotOpen database as ground truth, and MODIS reflectance data, vegetation indices, vegetation seasonality, land cover type, habitat heterogeneity, and topographical attributes as input features. These traits were subsequently used to derive global community-level CSR strategy signatures; 2) the cross-comparison of CSR functional strategy signatures with environmental constraints (e.g., stress data, disturbance data, and GPP product); 3) future projections of CSR functional strategy signatures using gradient boosting random forest models and WorldClim climate data and SoilGrids soil data.

**Figure S18** Principal component analyses (PCA) of eighteen community weighted mean (CWM) traits from sPlotOpen dataset (*n* = 95,104). These traits include leaf area (LA), stem specific density (SSD), specific leaf area (SLA), leaf carbon concentration (LCC), leaf nitrogen concentration (LNC), leaf phosphorus concentration (LPC), plant height (PH), seed mass (SM), seed length (SL), leaf dry matter content (LDMC), leaf nitrogen per area (LNPA), leaf N:P ratio (LNPR), leaf δ^15^N (LD15N), seed number per reproductive unit (SNRU), leaf fresh mass (LFM), stem conduit density (SCD), seed dispersal unit length (SDUL); and stem conduit element length (SCEL). The first two principal components (PCs) explain c. 51.51% of total trait variation. The colours represent CSR ternary strategies using a corresponding cyan-magenta-yellow (CMY) triplet colour composite, following the approach of Pierce, et al. ^32^.

**Figure S19** Global maps of three functional traits predicted using random forest models: specific leaf area (SLA, a), leaf dry matter content (LDMC, b), and leaf area (LA, c).

**Figure S20** Scatter plots comparing predicted and *in-situ* trait measurements from the testing dataset for SLA (a), LDMC (b), and LA (c). Blue lines represent the linear regression fits between the predicted and observed trait measurements. Dotted gray line represents the 1:1 line.

**Figure S21** Comparison of trait mapping for LDMC and SLA across seven plant functional types (PFTs) between the approach of Moreno-Martínez, et al. ^1^ and our method. These PFTs include evergreen needleleaf forest (ENF), evergreen broadleaf forest (EBF), deciduous needleleaf forest (DNF), deciduous broadleaf forest (DBF), shrublands (SHL), savannas (SAV), grasslands (GRL)).

**Figure S22** Density scatter plots comparing predicted and actual strategies derived from *in-situ* sPlotOpen trait measurements for C (a), S (b), and R (c) strategies. Red lines represent the linear regression fits between the predicted and actual strategies.

**References**

1	Moreno-Martínez, Á. *et al.* A methodology to derive global maps of leaf traits using remote sensing and climate data. *Remote Sensing of Environment* **218**, 69-88, doi:10.1016/j.rse.2018.09.006 (2018).

2	Sabatini, F. M. *et al.* sPlotOpen – An environmentally balanced, open‐access, global dataset of vegetation plots. *Global Ecology and Biogeography* **30**, 1740-1764, doi:10.1111/geb.13346 (2021).

3	Bruelheide, H. *et al.* sPlot – A new tool for global vegetation analyses. *Journal of Vegetation Science* **30**, 161-186, doi:10.1111/jvs.12710 (2019).

4	Kattge, J. *et al.* TRY plant trait database–enhanced coverage and open access. *Global Change Biology* **26**, 119-188 (2020).

5	Bruelheide, H. *et al.* Global trait–environment relationships of plant communities. *Nature Ecology & Evolution* **2**, 1906-1917, doi:10.1038/s41559-018-0699-8 (2018).

6	Friedl, M. A. *et al.* MODIS Collection 5 global land cover: Algorithm refinements and characterization of new datasets. *Remote sensing of Environment* **114**, 168-182 (2010).

7	Strahler, A. H. *et al.* MODIS BRDF/albedo product: algorithm theoretical basis document version 5.0. 42-47 (1999).

8	Huete, A. *et al.* Overview of the radiometric and biophysical performance of the MODIS vegetation indices. *Remote sensing of environment* **83**, 195-213 (2002).

9	Tucker, C. J. Red and photographic infrared linear combinations for monitoring vegetation. *Remote sensing of Environment* **8**, 127-150 (1979).

10	Xu, H. Modification of normalised difference water index (NDWI) to enhance open water features in remotely sensed imagery. *International journal of remote sensing* **27**, 3025-3033 (2006).

11	Rikimaru, A., Roy, P. & Miyatake, S. Tropical forest cover density mapping. *Tropical ecology* **43**, 39-47 (2002).

12	Tuanmu, M. N. & Jetz, W. A global, remote sensing‐based characterization of terrestrial habitat heterogeneity for biodiversity and ecosystem modelling. *Global Ecology Biogeography* **24**, 1329-1339 (2015).

13	Team, N. M. A. J. S. a. U. S. J. A. S. (distributed by NASA EOSDIS Land Processes DAAC, <https://doi.org/10.5067/ASTER/ASTGTM.003>, 2018).

14	Hijmans, R. J., Cameron, S. E., Parra, J. L., Jones, P. G. & Jarvis, A. Very high resolution interpolated climate surfaces for global land areas. *International Journal of Climatology: A Journal of the Royal Meteorological Society* **25**, 1965-1978 (2005).

15	Poggio, L. *et al.* SoilGrids 2.0: producing soil information for the globe with quantified spatial uncertainty. *Soil* **7**, 217-240 (2021).

16	Altman, J., Fibich, P., Trotsiuk, V. & Altmanova, N. Global pattern of forest disturbances and its shift under climate change. *Science of the total environment* **915**, 170117 (2024).

17	Li, X. & Zhou, Y. A Stepwise Calibration of Global DMSP/OLS Stable Nighttime Light Data (1992–2013). *Remote Sensing* **9**, 637 (2017).

18	Liu, X. *et al.* Annual dynamic dataset of global cropping intensity from 2001 to 2019. *Scientific Data* **8**, 283, doi:10.1038/s41597-021-01065-9 (2021).

19	Li, X. & Xiao, J. Mapping photosynthesis solely from solar-induced chlorophyll fluorescence: A global, fine-resolution dataset of gross primary production derived from OCO-2. *Remote Sensing* **11**, 2563 (2019).

20	Gorelick, N. *et al.* Google Earth Engine: Planetary-scale geospatial analysis for everyone. *Remote sensing of Environment* **202**, 18-27 (2017).

21	Breiman, L. Random forests. *Machine Learning* **45**, 5-32 (2001).

22	Hastie, T. *The elements of statistical learning: data mining, inference, and prediction*. (Springer, 2009).

23	Asner, G. P., Knapp, D. E., Anderson, C. B., Martin, R. E. & Vaughn, N. Large-scale climatic and geophysical controls on the leaf economics spectrum. *Proceedings of the National Academy of Sciences* **113**, E4043-4051, doi:10.1073/pnas.1604863113 (2016).

24	Pierce, S. *et al.* A global method for calculating plant CSR ecological strategies applied across biomes world-wide. *Functional Ecology* **31**, 444-457, doi:10.1111/1365-2435.12722 (2017).

25	Díaz, S. *et al.* The global spectrum of plant form and function. *Nature* **529**, 167-171 (2016).

26	Campbell, B. D. & Grime, J. P. An experimental test of plant strategy theory. *Ecology* **73**, 15-29 (1992).

27	Li, Y. & Shipley, B. An experimental test of CSR theory using a globally calibrated ordination method. *PLoS One* **12**, e0175404 (2017).

28	Guo, K. *et al.* Stage dependence of Elton’s biotic resistance hypothesis of biological invasions. *Nature Plants* **10**, 1484-1492, doi:10.1038/s41477-024-01790-0 (2024).

29	Guo, K. *et al.* Plant invasion and naturalization are influenced by genome size, ecology and economic use globally. *Nature Communications* **15**, 1330, doi:10.1038/s41467-024-45667-4 (2024).

30	Hodgson, J., Wilson, P., Hunt, R., Grime, J. & Thompson, K. Allocating CSR plant functional types: a soft approach to a hard problem. *Oikos* **85**, 282-294 (1999).

31	Sabatini, F. M. *et al.* Global patterns of vascular plant alpha diversity. *Nature Communications* **13**, 4683, doi:10.1038/s41467-022-32063-z (2022).

32	Pierce, S., Luzzaro, A., Caccianiga, M., Ceriani, R. M. & Cerabolini, B. Disturbance is the principal α‐scale filter determining niche differentiation, coexistence and biodiversity in an alpine community. *Journal of Ecology* **95**, 698-706, doi:10.1111/j.1365-2745.2007.01242.x (2007).
